# VEGF mediates fat embolism-induced acute lung injury via VEGF receptor 2 and the MAPK cascade

**DOI:** 10.1038/s41598-019-47276-4

**Published:** 2019-08-12

**Authors:** Chin-Kuo Lin, Yu-Hao Lin, Tai-Chun Huang, Chung-Sheng Shi, Cheng-Ta Yang, Yi-Ling Yang

**Affiliations:** 1 Division of Pulmonary Infection and Critical Care, Department of Pulmonary and Critical Care Medicine, Chang Gung Memorial Hospital, Chiayi, Taiwan; 2 Graduate Institute of Clinical Medicine Sciences, College of Medicine, Chang Gung University, Taoyuan, Taiwan; 3 Department of Biochemical Science and Technology, National Chia-Yi University, Chia-Yi, Taiwan; 4 Department of Life Science, National Taiwan Normal University, Taipei, Taiwan; 5 Division of Colon and Rectal Surgery, Department of Surgery, Chang Gung Memorial Hospital, Chiayi 61363, Taiwan; 6 Department of Pulmonary and Critical Care Medicine, Chang Gung Memorial Hospital, Taoyuan, Taiwan; 7 Department of Respiratory Care, College of Medicine, Chang Gung University, Taoyuan, Taiwan

**Keywords:** Respiration, Experimental models of disease

## Abstract

Fat embolism (FE) is a lethal medical emergency often caused by fracture of long bones and amputation of limbs. Vascular endothelial growth factor (VEGF) promotes angiogenesis and increases vascular permeability. We tested the hypothesis that VEGF plays a critical role in FE-induced acute respiratory distress syndrome (ARDS) and acute lung injury (ALI). Fat tissues were collected from male Sprague-Dawley rats, and animal oil was extracted and mixed with water to form fatty micelles. The micelles were then injected into the tail vein to produce FE and ALI in rats. Lung weight gain was measured as the index of pulmonary edema. The expression of pulmonary VEGF was evaluated by real-time PCR and western blot analysis. Inducible nitric oxide synthase (iNOS) and phosphorylation of mitogen-activated protein kinase (MAPK) were determined by western blot analyses. Interleukin-1β (IL-1β) was quantified by ELISAs. Hematoxylin and eosin staining was used to evaluate the pathological damage of ALI. In this study, we found that animal oil-induced FE significantly increased pulmonary VEGF expression and MAPK phosphorylation. We also evaluated the inflammatory response after FE and found that iNOS and IL-1β significantly increased after FE. Systemic administration of SU-1498, an antagonist of VEGF receptor 2 (VEGFR-2), significantly attenuated the FE-induced inflammatory response and histological damage. This study suggested that VEGF is involved in FE-induced ARDS via the VEGFR-2 and MAPK cascades, which induce IL-1β release and iNOS upregulation. Blockade of could be used to treat FE-induced pulmonary damage.

## Introduction

Fat embolism (FE) is a serious clinical complication in patients associated with long-bone fractures or amputation. Intravasation of fat or fatty acids from long-bone fractures and other sources leads to FE, which always induces severe lung injury and acute respiratory distress syndrome (ARDS)^1,2^. Circulating fat emboli cause microvascular obstruction presenting the initial physical signs; subsequently, the products of hydrolyzed neutral fat by lipase are toxic to the lung^3^. Release of various chemical mediators causes acute lung injury (ALI). Plasma serotonin, nitrate/nitrite, methyl guanidine, which is an index of free radicals, tumor necrosis factor α (TNF-α), interleukin-1β (IL-1β), and interleukin-10 (IL-10) were shown to be significantly elevated in FE-induced ARDS patients^4–7^, and inhibition of iNOS could significantly attenuate the FE-induced inflammatory response and pulmonary damage^7–9^.

Vascular endothelial growth factor (VEGF) stimulates endothelial cell growth *in vivo* and *in vitro* and is a member of the family of hypoxia-inducible proteins that interact with the receptor tyrosine kinases on endothelial cells. VEGF family members, including VEGF-A, VEGF-B, VEGF-C, VEGF-D, and the placental growth factor^10,11^, which are closely related to angiogenesis and promote vasopermeability, wound repair and tumor growth^12–15^. The main mediator of angiogenesis is the dimeric glycoprotein VEGF-A, which is usually referred to as VEGF. VEGF functions as a potent proinflammatory cytokine in many physiological and pathological immune responses^16^. Numerous signals, such as IL-1, IL-6, and insulin-like growth factor I, are known to stimulate VEGF transcription^17^. There are four subtypes of VEGF receptors (VEGFR): VEGFR-1, VEGFR-2, VEGFR-3, and the neuropilin receptor. The major VEGFR involved in angiogenesis has been shown to be VEGFR-2^18,19^. The levels and activities significantly increase along with the VEGF level under pathological conditions^20,21^. Plasma VEGF levels and its receptors were shown to increase under severe sepsis conditions, and the VEGF levels correlated with the severity and mortality, which are primarily mediated through the nitric oxide (NO) signaling pathway^22–25^.

The impaired ventilation/perfusion ratio after FE leads to hypoxemia, which is considered the most common earliest symptom of this syndrome. Hypoxia is a strong stimulus for angiogenesis; when cells suffer hypoxia, they release angiogenic factors to re-establish oxygen supply through vessel formation^26^. Our previous studies suggested that VEGFR-2 mediated the effects of VEGF via the MAPK cascade and played a critical role in neuronal damage^27^. In this study, we evaluated the role of VEGF in FE-induced ARDS and its possible mechanisms.

## Results

### VEGF expression in lung tissues significantly increased after fat embolism

**Figure 1:**
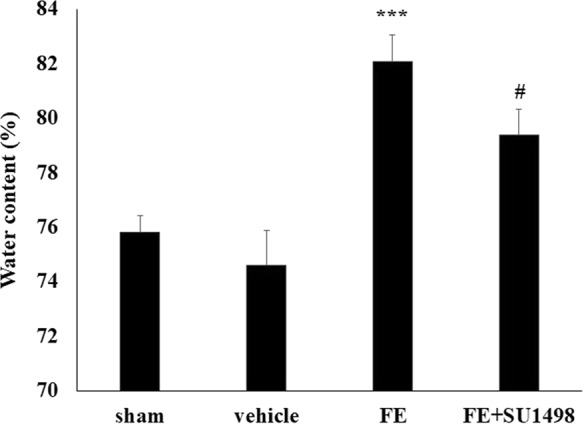
Effect of FE and VEGF receptor 2 antagonist SU1498 administration on pulmonary edema. Here, we use the LWG ratio as an index of pulmonary edema. Compared with the sham group, FE induced severe pulmonary edema, and the water content increased from 75.8% ± 0.59% to 82.1% ± 0.97%. Administration of SU1498 significantly attenuated the FE-induced pulmonary edema to 79.4% ± 0.92%. Data are represented as the mean ± SEM values. ***p < 0.001 was considered significantly different from sham values, ^#^*p* < 0.05 was considered significantly different from the value of the FE group by a Mann–Whitney U-test.

**Figure 2:**
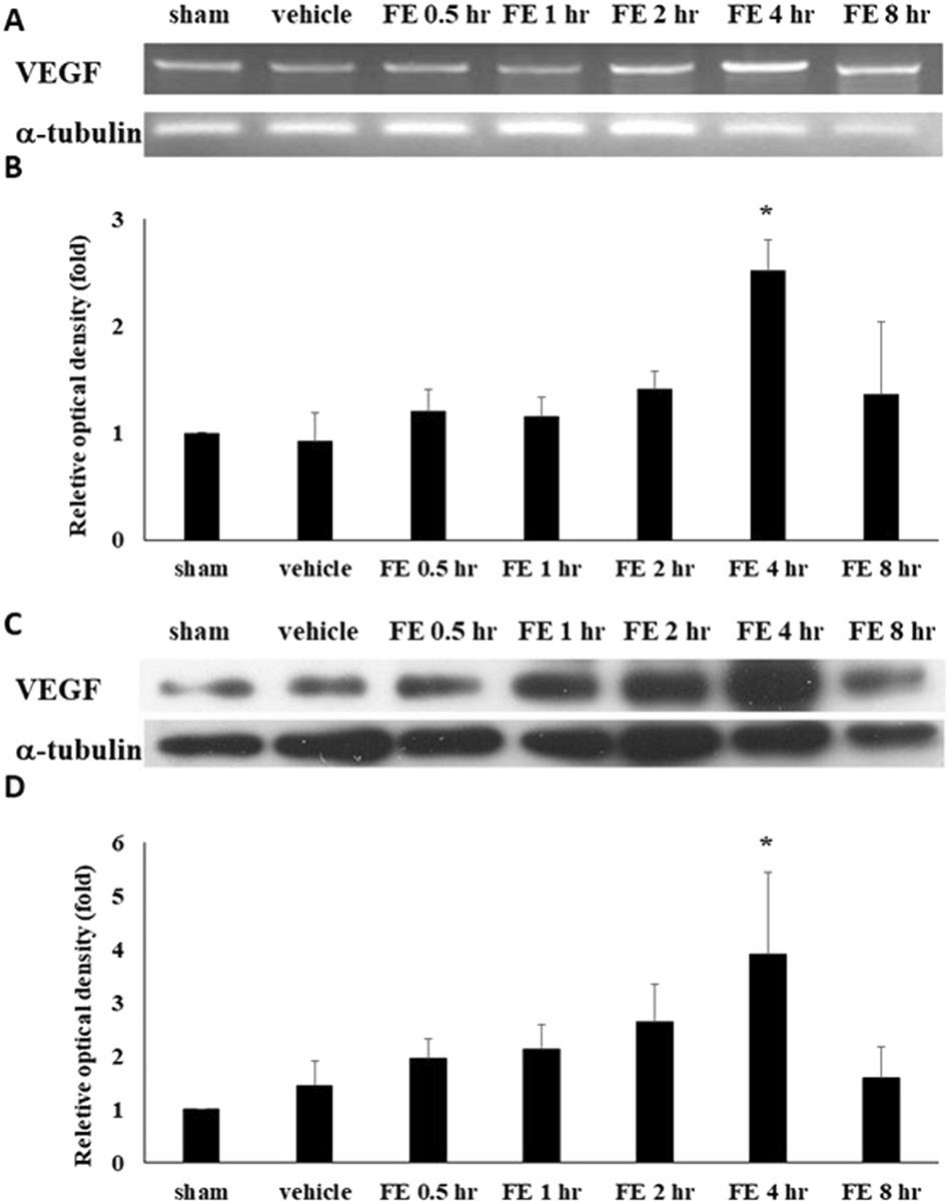
Expression of VEGF in lung tissue after FE. (**A**,**B**) Expression and quantification of VEGF mRNA after TBI. The numbers above the top panel indicate hours after injury. (**C**,**D**) Expression and quantification of VEGF protein after TBI. Relative density presented as a percent increase compared with the sham group. Semiquantitative densitometry in conjunction with AlphaEase software was used for the quantification, and the data are presented as ratios to control values. Compared with the sham group rats (with no FE induction), both VEGF mRNA and protein significantly increased at 4 hours after FE in the lung and returned to basic levels at 8 hours after FE induction. Bars represent the mean ± SEM values. *p < 0.05 was considered significantly different from sham values by Mann–Whitney U-tests.

The FE-induced lung edema was evaluated using lung weight gain (LWG). Compared with the sham group animals (without FE induction), FE induced severe pulmonary edema, and the water content significantly increased from 75.8% ± 0.59% to 82.1% ± 0.97% (Fig. 1). In addition, both VEGF mRNA and protein in lung tissues significantly increased at 4 hours after FE in the lungs and were back to baseline levels at 8 hours after FE induction (Fig. 2). This finding suggests that VEGF might be involved in FE-induced lung edema.

### VEGF receptor 2 mediates FE-induced pulmonary edema

**Figure 3:**
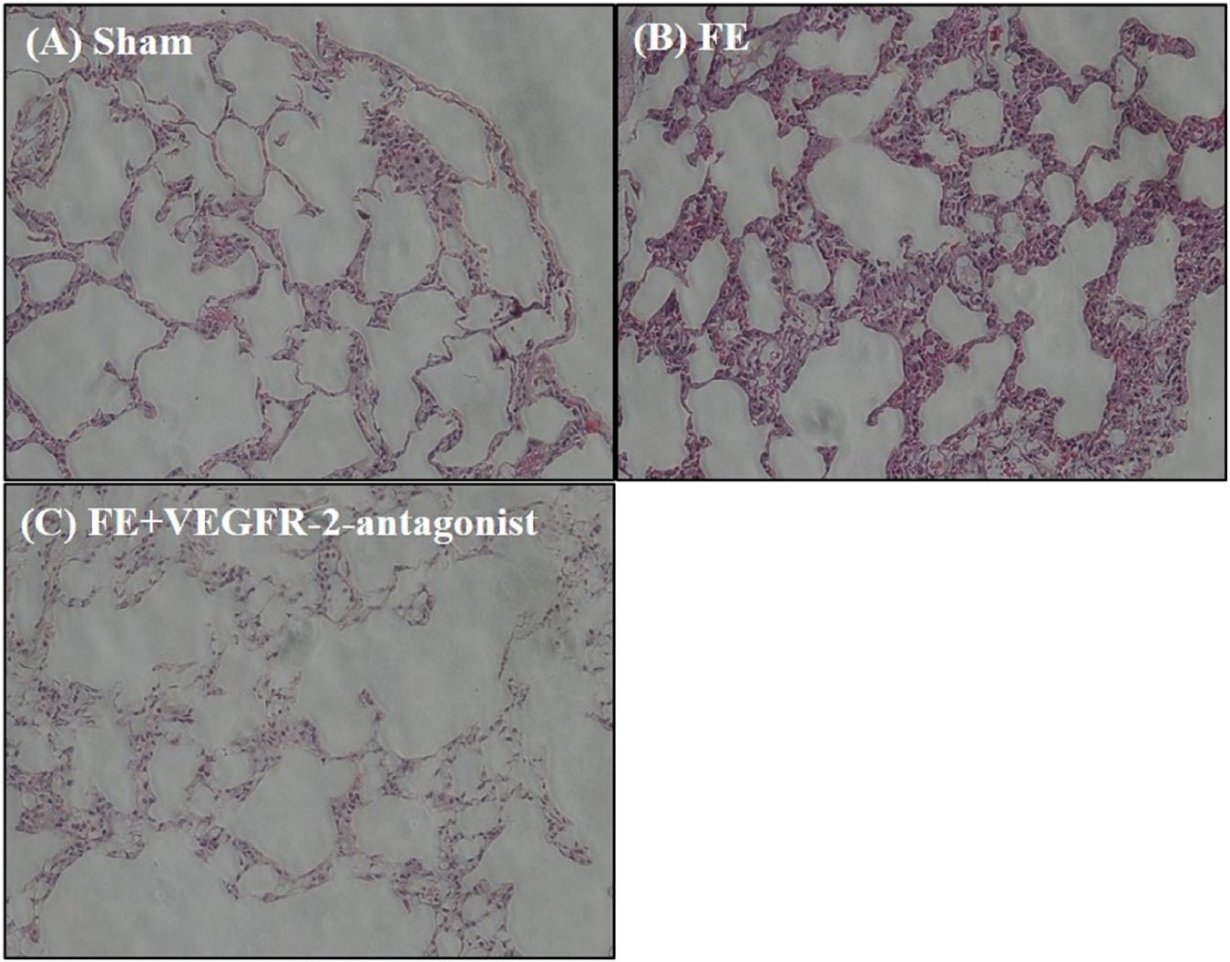
Photomicrographs showing alveolar morphology in sham rats (**A**), rats with fat embolism (FE) (**B**), or rats with fat embolism and treated with SU1498 (**C**). After fixation, coronal sections (5 μm) through the alveoli were stained with hematoxylin and eosin for microscopic evaluation. Compared with the control group (**A**), FE induced severe histological damage, which showed infiltration of cells. Compared with the FE group, administration of SU1498 significantly alleviated the histological injury after FE treatment.

The functional role of VEGF in FE-induced edema was further examined by SU1498, an antagonist of VEGF receptor 2 (VEGFR-2). Our previous study showed that VEGFR-2 plays an essential role in traumatic brain injury-induced brain edema (Lu *et al*., 2015). We speculated that a similar mechanism might be involved here. The FE-induced severe pulmonary edema was significantly attenuated after systemic administration of SU1498 (0.4 mg/kg, I.V.) from 82.1% ± 0.97% to 79.4% ± 0.92%, which suggested that VEGFR-2 mediated the effect of VEGF on FE-induced pulmonary edema (Fig. 1). The possible rescue effect of SU-1498 on FE-induced pulmonary damage was also evaluated by hematoxylin and eosin staining. Compared with the FE group, administration of SU-1498 significantly alleviated the FE-induced pulmonary damage (Fig. 3). These results strengthened our hypothesis that VEGF participated in FE-induced pulmonary edema and subsequent pulmonary injuries via activation of VEGFR-2.

### The MAPK cascade is involved in the VEGF receptor 2 pathway after FE

**Figure 4:**
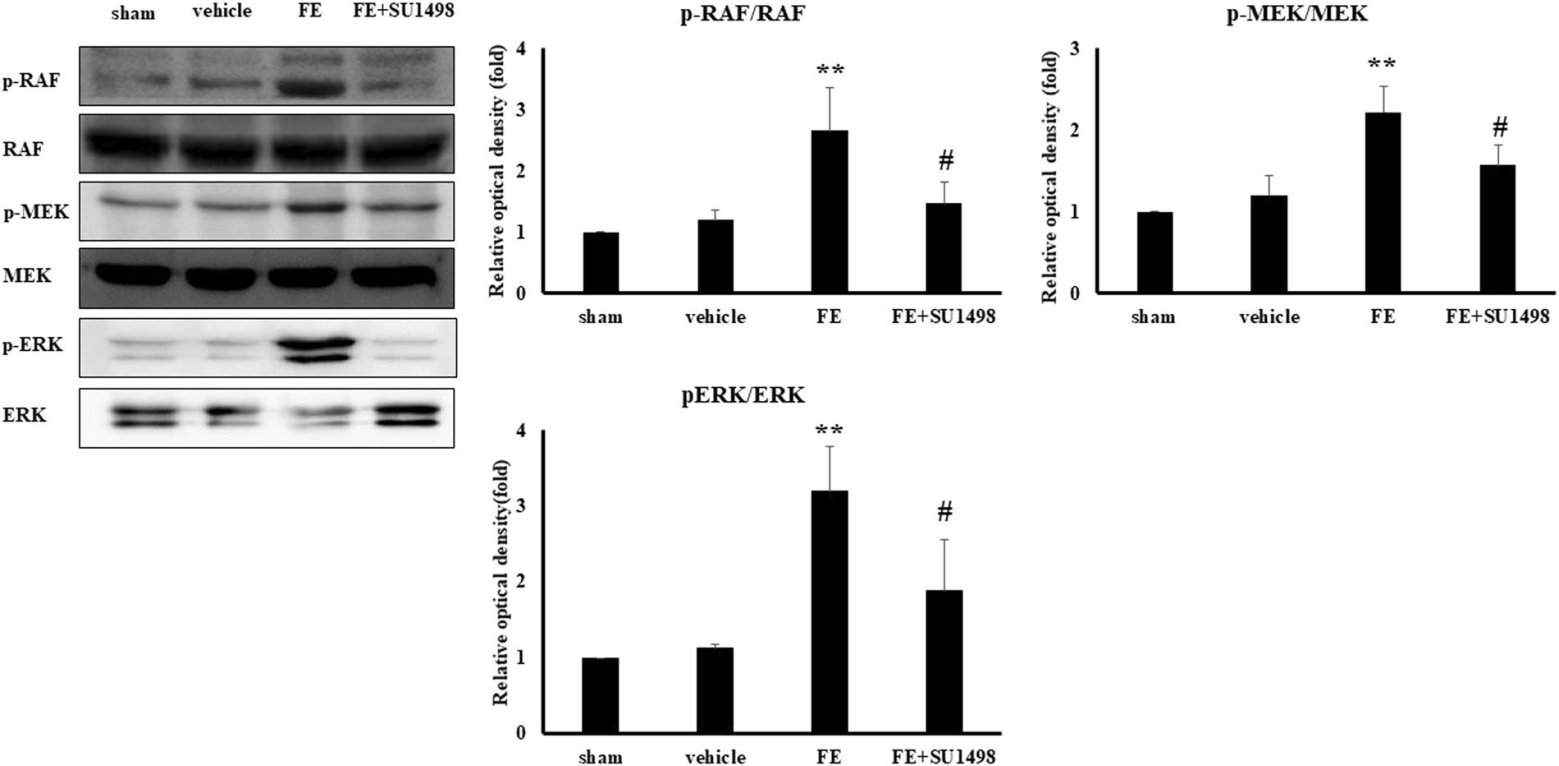
Effects of SU1498 on the pulmonary phosphorylation of Raf (p-Raf), MEK (p-MEK), and ERK (p-ERK) in sham rats (sham), rats with vehicle (vehicle), rats with fat embolism (FE), or rats treated with SU1498 (FE + SU1498). Left: western blot analysis of sham rats (sham), rats with vehicle (vehicle), rats with fat embolism (FE), or rats treated with SU1498 (FE + SU1498); Right: Relative density presented as percent increase compared with sham group. Data are represented as the mean ± SEM values (n = 5). **p < 0.01 was considered significantly different from sham values, and ^#^*p* < 0.05 was considered significantly different from rats with FE treatment by the Mann–Whitney U-test.

The MAPK cascade, which includes Raf, MEK and ERK, plays a critical role in VEGFR-2 activation after brain trauma^27^. Therefore, the MAPK cascade became our prime target for investigation. To elucidate the possible involvement of the MAPK cascade in FE-induced lung edema, we analyzed the expression of pulmonary Raf, MEK and ERK phosphorylation after FE. We found that MAPK cascade phosphorylation increased after FE. Administration of a VEGFR-2 antagonist (SU-1498) decreased MAPK cascade phosphorylation after FE (Fig. 4), suggesting that MAPK cascade phosphorylation is involved in VEGFR-2 activation after FE.

### FE-induced overexpression of iNOS and IL-1β and blockade of VEGFR-2 attenuated the inflammatory response after FE

**Figure 5:**
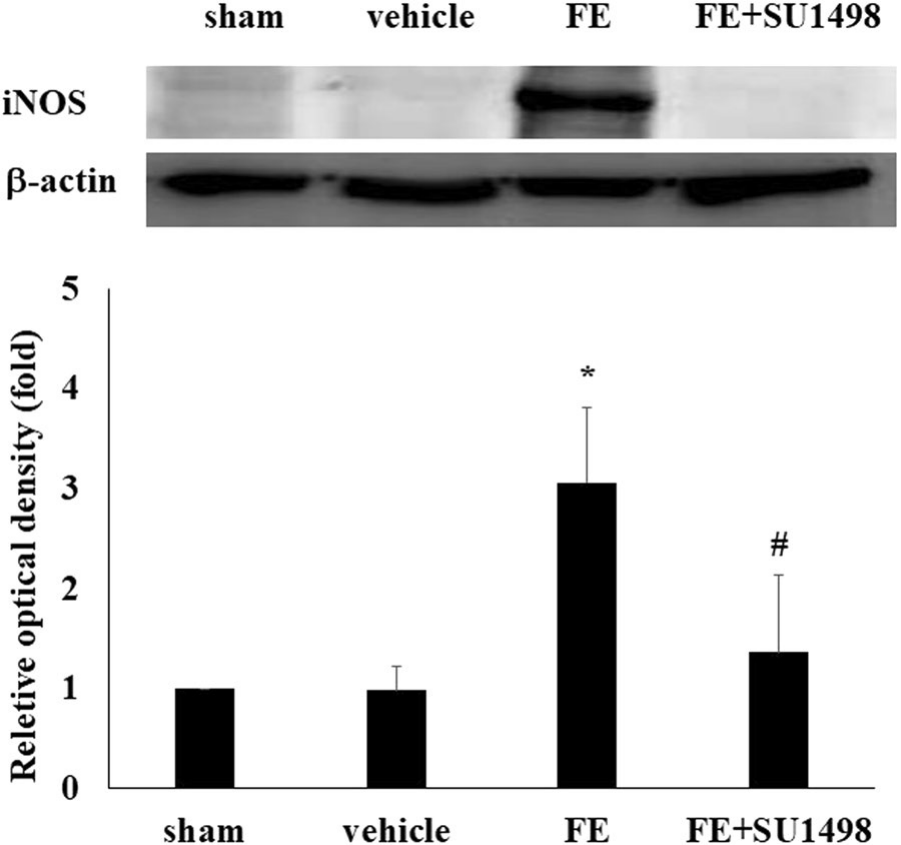
Effects of SU1498 on pulmonary iNOS expression in sham rats (sham), rats with vehicle (vehicle), rats with fat embolism (FE), or rats treated with SU1498 (FE + SU1498). Relative density presented as percent increase compared with the sham group. Data are represented as the mean ± SEM values. *p < 0.05 was considered significantly different from sham values, and ^#^*p* < 0.05 was considered significantly different from rats with FE treatment by the Mann-Whiney U test.

**Figure 6:**
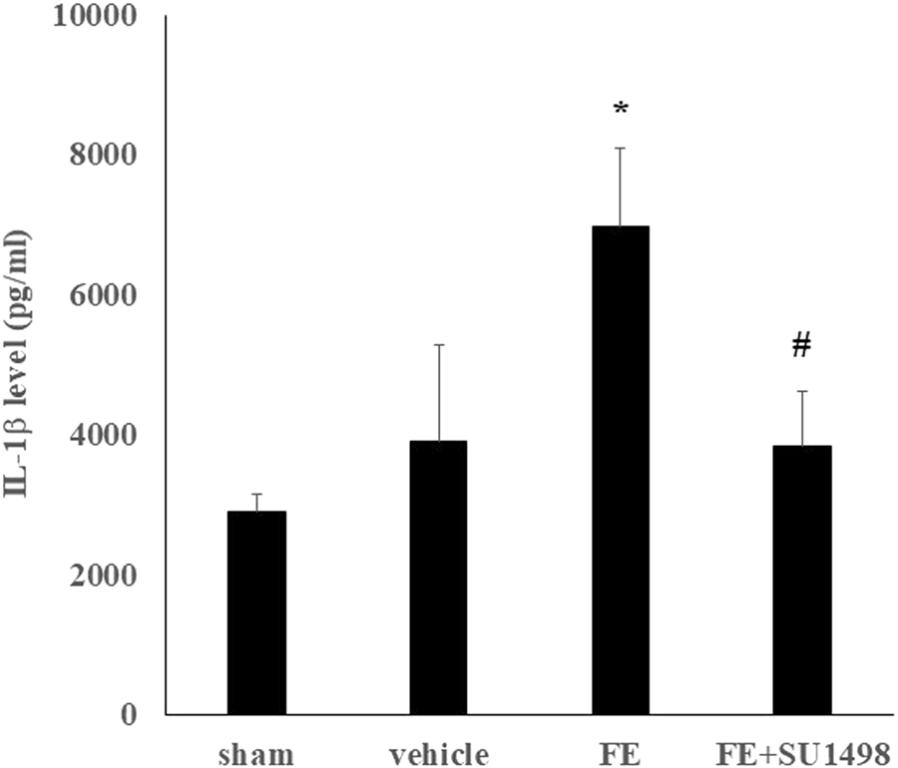
Effects of FE and SU1498 administration on the production of IL-1β by ELISA. The relative level of the released IL-1β is compared among differently treated animal groups: sham; vehicle, FE; FE combined with SU1498 injection (FE + SU1498). IL-1β was significantly increased after FE, and SU1498 administration significantly alleviated IL-1β overproduction. Bars represent the mean ± SEM values. *p < 0.05 is considered significantly different from the sham value, and ^#^*p* < 0.05 is considered significantly different from the FE value by the Mann-Whiney U test.

Recent research has shown that inflammatory mediators play a key role in the pathogenesis of ARDS^28^. Biochemical data also indicated that the concentrations of nitrite and nitrate are increased in bronchoalveolar lavage fluid after FE^29^. The level of inducible NOS (iNOS) is an important index of the inflammatory response and nitrate level. After FE, the expression of iNOS was significantly increased (3-fold) but was significantly attenuated by SU-1498 administration (1.3-fold) (Fig. 5). To further investigate the role of FE-induced pulmonary inflammation and the effects of SU-1498 on the FE-induced inflammation response, IL-1β expression in pulmonary lavage was measured by ELISAs. The results suggested that IL-1β expression was significantly increased after FE and VEGFR-2 antagonist administration significantly alleviated IL-1β overproduction (Fig. 6).

## Discussion

The present study clearly demonstrated that FE induced severe pulmonary edema and damage, which is mediated by overexpression of VEGF, and then activated the VEGFR-2 and MAPK cascades. Our results also suggested that FE induced a pulmonary inflammatory response, which included IL-1β and iNOS overproduction. Most importantly, either FE-induced pulmonary edema or the inflammatory response could be rescued by VEGFR-2 antagonist administration. These results suggested that VEGF and VEGFR-2 play a critical role in FE-induced pulmonary damage. The VEGFR-2 antagonist may be a potential target for developing therapeutic agents of FE-induced pulmonary edema and damage.

Acute respiratory distress syndrome (ARDS) is the major lethal complication of FE. This syndrome results from an acute, diffuse injury to the alveolar-capillary wall, which is responsible for respiratory failure. ARDS caused interstitial and alveolar edema by increasing the capillary and alveolar permeability. The endothelial permeability increase is thought to involve both neutrophil-dependent and neutrophil-independent mechanisms^30^. VEGF is an angiogenic and permeability-increasing factor that is secreted by almost all the cell types found in airspaces or their lining during ARDS, namely, alveolar epithelial cells, macrophages, and polymorphonuclear neutrophils. Since we used qPCR and western blotting to evaluate the change in VEGF expression after FE, the responsible cell type(s) was not yet determined. Future studies using immunohistochemistry or *in situ* hybridization will reveal the cell types involved in the elevated VEGF expression. In this study, we found that overexpression of pulmonary VEGF played an important role in FE-induced pulmonary damage, which may account for the development of ARDS. This finding was consistent with previous studies suggesting that VEGF expression is elevated in various ARDS models, such as acid-induced lung injury or lipopolysaccharide-induced murine lung injury. In ARDS patients, the VEGF levels were significantly greater in non-survivors than survivors of ARDS. Furthermore, changes in VEGF appear to be associated with either a good outcome if VEGF levels fall (12% subsequent mortality) or a bad outcome if VEGF levels rise (78% mortality). Increases in plasma VEGF of over 100% baseline values were associated with 100% mortality^31^. In the present study, we provided direct evidence of the involvement of VEGF in FE-induced pulmonary damage. Our results suggest that the VEGF level may be a biomarker of the severity of FE-induced pulmonary damage or survival rate and therapeutic agent targets.

However, the pathological role of VEGF in acute lung injury (ALI) and ARDS remains controversial. Compared with the patients who died after FE, the VEGF level in lung epithelial lining fluid (ELF) was significantly increased in the patients who survived from FE, and the VEGF level in ELF was also inversely correlated with lung injury score (LIS)^32^. Furthermore, the levels of VEGF were low in ARDS patients with a very high mortality rate, and an increase in endothelial cell apoptosis and a decrease in capillary density were found^33^. Thickett *et al*. reported that increased VEGF levels in ELF on day 4 were associated with recovery from ALI/ARDS and concluded that VEGF levels in the alveolar space may predict recovery from ALI^34^. VEGF protects endothelial cells against apoptosis^35^ and induces endothelial cell proliferation and contributes to endothelial barrier regeneration. In the present study, only short-term changes in pulmonary VEGF expression (within 8 hours after FE) were observed, and the VEGFR-2 antagonist was administered immediately after FE. We cannot exclude the possibility that VEGF may play a protective role against FE-induced lung injury.

Although systemic administration of a VEGFR-2 antagonist attenuated the severity of FE-induced lung edema and injury, the possibility of other VEGFRs cannot be eliminated. Several mechanisms are responsible for the regulation of VEGF levels. For instance, the soluble form VEGFR-1 (sVEGFR-1) is a splice variant of membrane-bound VEGF receptor 1 (VEGFR-1)^36^, which is a potent antagonist of VEGF. sVEGFR-1 also appeared in the ELF of ARDS patients^37^, and the addition of recombinant sVEGFR-1 significantly abolished the VEGF-induced increase in pulmonary artery endothelial cell monolayer permeability^34^. In our study, we found that VEGFR-2 is critical to ARDS-induced pulmonary effects via the MAPK cascade. SU-1498 is a specific VEGFR-2 antagonist that significantly attenuates FE-induced pulmonary inflammation and cell damage. This result is consistent with the previous observation of a disintegrin and metallopeptidase with thrombospondin type 1 motif 1 (ADAMTS-1), an extracellular matrix protease. ADAMTS-1 inhibits VEGF function by reversibly binding with VEGF and blocking VEGFR-2 phosphorylation, leading to the suppression of endothelial cell proliferation^38^. Further studies, such as evaluation of the expression of sVEGFR-1 and ADAMTS1, are necessary to elucidate the role of other VEGF receptors in FE-induced pulmonary damage.

We provided a linkage between VEGF and inflammation signals, such as iNOS and IL-1β, in FE-induced lung injury. The expression of iNOS and IL-1β was significantly increased and was significantly attenuated by SU1498 administration. We speculate that shortly after FE, oily vacuoles inside alveolar capillaries induced acute inflammatory exudate in alveolar sacs with hyaline membranes and macrophages. This histopathology is associated with an impaired ventilation/perfusion ratio, resulting in hypoxemia, which is the most common earliest symptom of FE-induced lung injury. Hypoxia activates hypoxia-inducible transcription factors (HIFs), which are known to promote the expression of VEGF transcriptionally by binding to a hypoxia response element (HRE) in the VEGF promoter. In addition to VEGF, iNOS and IL-1β are also targets of HIF-1α^39^, indicating that hypoxia after FE induces a chain reaction that exacerbates the inflammatory response and lung damage. Inflammatory cytokines can reciprocally regulate the HIF pathway. For example, IL-1β and TNF-α could activate HIF-1α expression via the MAPK cascade and phosphoinositide 3-kinase (PI3K)^40–42^, which suggested that VEGF and iNOS were also upregulated via HIF-1α activation. Further experiments could focus on the expression and regulation of HIFs to reveal their involvement in FE-induced lung injury. In addition, inflammatory cytokines can directly induce VEGF expression via the ERK- and p38-dependent pathway^40,43^. Activation of p38 is reported to upregulate the expression of cyclooxygenase-2 (COX-2), which could directly induce VEGF upregulation^44^. Collectively, these results suggested that hypoxia induced VEGF expression and cytokine release, which exacerbated FE-induced pulmonary damage in a synergistic manner. Blocking this vicious circuit will be an essential strategy in FE treatment. In addition, FDA-approved VEGFR2 antagonists such as Cyramza (ramucirumab) are specifically indicated for advanced gastric cancer or gastro-esophageal junction adenocarcinoma. FDA-approved VEGFR2 antagonists should be further evaluated.

In conclusion, VEGFR-2 blockade significantly attenuated pulmonary damage after FE via inhibition of MAPK phosphorylation, IL-1β release, and iNOS overproduction. Our results suggest the therapeutic potential of a VEGFR-2 antagonist on FE-induced pulmonary damage.

## Material and Methods

### Fat embolism induction

Male Sprague-Dawley rats, weighing 350–370 g were purchased from BioLASCO, Taiwan, Co., Ltd. and housed individually in a temperature-controlled animal colony at 24 °C, with a normal 12-hrs:12-hrs light/dark cycle. The animals had free access to food and water, and allowed to acclimate to the light/dark cycle at room temperature for at least 1 week before undergoing the experiments. Fat tissues were collected from rats, heated to extract the animal oil and then filtered and removed from the debris. The animal oil was then mixed with water (1:1) to form fatty micelles. The micelles (0.2 ml) were injected into the tail vein to induce FE and acute lung injury in rats. All procedures were conducted in accordance with the National Institutes of Health Guide for Care and Use of Laboratory Animals and all the protocols were approved by the institutional animal care and use committee (IACUC) at the National Chia-Yi University (IACUC Approval Number: 105031). All efforts were made to minimize animal suffering and to minimize the number of animals necessary to produce reliable data.

### Pulmonary edema evaluation

The lung weight gain ratio (LWG) was used as an index of pulmonary edema and ALI, and a higher ratio suggests more severe pulmonary edema and damage. The lungs were removed after FE was weighed as the initial LW, and the lungs were dried in an oven at 120 °C for 48 hours to obtain the final lung weight. The LWG was obtained from the following equation: LWG% = (final LW-initial LW)/initial LW*100%^45,46^.

### Western blot: Determine the expression of pulmonary VEGF, iNOS and the phosphorylation of mitogen-activated protein kinases

The lung tissues were weighed and rapidly thawed in 6 volumes of ice-cold homogenizing buffer containing protease inhibitor cocktail (Thermo Scientific, UT, USA #78430) to prevent protein degradation. Then, the samples were homogenized with a sonicator, and T-PER tissue protein extraction reagent (Thermo Scientific, UT, USA #78510) was used to isolate nuclear protein. Following electrophoretic separation by 10% sodium dodecyl sulfate (SDS) gel, the resolved proteins were transferred electrophoretically to a polyvinylidene difluoride membrane. Antibodies including anti-VEGF, anti-MAPK cascade (ERK, Raf and Ras) and anti-iNOS were used, and bound antibody was visualized with an enhanced chemiluminescence assay (ECL) (Bio Kit Biotechnology, Inc., Taiwan). The PVDF membranes were first incubated in primary antibody TBST solution overnight at 4 °C. The membranes were then probed with HRP-conjugated secondary antibodies for 1 h at room temperature. The signals were analyzed using a Quantity One digital imaging system (Bio-Rad, USA). The expression levels of VEGF and iNOS were determined using the expression relative to that of α-tubulin or β-actin, and the expression levels of p-Raf, p-MEK and p-ERK were determined by the expression relative to RAF, Raf and Ras, respectively. The relative optical density was adjusted in each case for the respective internal control and sham control.

### Real-time polymerase chain reaction: detect elevated VEGF expression after FE

In this study, we determined the transcript level of VEGF by real-time PCR. Total hippocampal RNA was extracted with TRIzol reagent (Gibco BRL, Grand Island, NY, USA), and total RNA was isolated according to the manufacturer’s protocol. The integrity of RNA was quantified by spectrophotometry (Thermo Scientific, UT, USA). Samples with OD260/280 (1.8–2.0) were used for further expression level detection. In brief, real-time PCR was performed according to the manufacturer’s protocol of the TaqMan RT kit and TaqMan assay kit (Applied Biosystems, USA).

### Enzyme-linked immunosorbent assay: measure IL-1β level

After FE or drugs administration, animals were decapitated and their lungs were collected, weighed and rapidly thawed in 6 volumes of ice-cold homogenizing buffer. Then the samples were homogenized with a sonicator. The level of IL-1β in pulmonary tissue was determined by ELISA (Abcam, UK, ab100768) in accordance with the instructions of the manufacturer.

### Hematoxylin and eosin staining: evaluation of lung injury

After fat embolism induction, each group of rats was sacrificed by an overdose of pentobarbital (100 mg/kg, intraperitoneally) and then perfused transcardially with 0.9% NaCl and 10% formalin. After perfusion, the rats were decapitated, and their pulmonary tissue was collected and embedded in paraffin blocks. Slice sections (5 µm thickness) were stained with hematoxylin and eosin (H&E) and subjected to microscopic examination. The pulmonary damage was evidenced by counting the infiltration of immune cells.

### Statistical analysis

All data, including protein levels from immunoblotting are presented as mean standard error of the mean (SEM). Either nonparametric Mann-Whitney U test or analysis of variance (ANOVA) with Bonferroni-Dunn post-hoc testing was used, and a p value < 0.05 was considered statistically significant.
